# SIRT1 Deacetylates TET2 and Promotes Its Ubiquitination Degradation to Achieve Neuroprotection Against Parkinson's Disease

**DOI:** 10.3389/fneur.2021.652882

**Published:** 2021-04-15

**Authors:** Xuan Li, Te Liu, Ting-Ting Wu, Ya Feng, Si-Jia Peng, Huiyong Yin, Yun-Cheng Wu

**Affiliations:** ^1^Department of Neurology, Shanghai General Hospital, Shanghai Jiao Tong University School of Medicine, Shanghai, China; ^2^Shanghai Geriatric Institute of Chinese Medicine, Shanghai University of Traditional Chinese Medicine, Shanghai, China; ^3^CAS Key Laboratory of Nutrition, Metabolism and Food Safety, Shanghai Institute of Nutrition and Health, Chinese Academy of Sciences, Shanghai, China; ^4^Key Laboratory of Food Safety Risk Assessment, Ministry of Health, Beijing, China; ^5^School of Life Science and Technology, Shanghai Tech University, Shanghai, China

**Keywords:** Ten-Eleven Translocation 2, Silence information regulator 1, resveratrol, cyclin dependent kinase inhibitor 2A, Parkinson's disease

## Abstract

The epigenetic modifications, such as DNA methylation and histone acetylation, play a critical role in the pathogenesis of Parkinson's disease (PD). However, the relationship between DNA methylation and histone acetylation in PD is not fully understood. Previous studies have shown that patients with PD exhibit an epigenetic and transcriptional upregulation of Ten-Eleven Translocation 2 (TET2), a member of the DNA hydroxylases family. Silence information regulator 1 (SIRT1), a nicotinamide adenine dinucleotide (NAD)-dependent histone deacetylase, also plays a critical role in PD development and might be a potential target for PD therapy. Our previous data indicated that demethylation in the Cyclin-dependent kinase inhibitor 2A (CDKN2A) promoter by the TET2 directly activated its expression, then promoted the cell cycle arrest and cell death induced by 1-methyl-4-phenyl-pyridinium ion (MPP^+^). In this study, we found that the enzyme activity of SIRT1 is negatively correlated with the protein level of TET2. In addition, the deacetylation of TET2 induced by SIRT1 promotes TET2 degradation via the ubiquitin–proteasome pathway. Furthermore, the activation of endogenous SIRT1 by resveratrol (RV) leads to CDKN2A DNA hypermethylation due to the decreased TET2 protein levels, which relieves the inhibitory effect on CDK4 and upregulation of pRb, allowing cell proliferation and growth. Similar effects are observed for the inhibition of endogenous TET2 enzyme activity with TET2 inhibitor. Together, we discover a new mechanism by which the SIRT1-TET2-CDKN2A pathway is involved in the pathogenesis of PD, which may provide a potential target for PD treatment.

## Highlights

(1) Deacetylation of TET2 induced by SIRT1 promotes TET2 degradation via the ubiquitin–proteasome pathway.
(2) The CDKN2A/p16INK4a-CDK4-pRB signaling pathway is involved in the effect of TET2 on the cell cycle in the PD cellular model.

## Introduction

Parkinson's disease (PD) is the second most common neurodegenerative disease, affecting about 1.0% of the population over 60 years of age and 3.0% of the population over 80 years of age (1). The pathogenesis of PD includes mitochondrial dysfunction, oxidative stress, inflammation, genetic factors, epigenetic modifications, etc. (2). However, the causative factors of neurodegenerative process affecting the etiopathogenesis of PD remain unknown (3).

Idiopathic PD is mediated by mutations in genes such as α-synuclein, parkin, PTEN-induced kinase 1, and leucine-rich repeat kinase 2, protein DJ-1, but these account for only about 10% of PD patients (4). The majority of PD cases are sporadic, which is currently conceptualized as a consequence of various genetic variants and complex environment-gene interactions on a background of age-related changes adding up to individual levels of susceptibility (5). DNA modifications, particularly DNA methylation, show the most promise for epigenetic biomarker development in neurodegenerative diseases recently. Given the known influence of α-synuclein expression levels in PD, initial DNA methylation studies focused on the SNCA gene. It has been reported that the methylation of human SNCA intron 1 decreased gene expression while the inhibition of DNA methylation activated its expression in the brains of PD patients (6). Although postmortem brain analysis revealed regional non-specific methylation differences in this CpG region in the anterior cingulate and putamen among controls and PD, methylation was significantly decreased in the substantia nigra of PD patients (7). The results suggest that a decreased DNA methylation or increased demethylation maybe involved in the pathogenesis of PD.

DNA demethylation relies on the ten-eleven translocation (TET) enzyme to catalyze the conversion of DNA methylation to hydroxymethylation, which has also been shown to affect neurodevelopment and synaptic transmission (8). In mammals, the TET enzyme family includes TET 1, 2, 3. They have the same catalytic activity and can reverse DNA methylation by oxidizing 5-methylcytosine (5mC) to 5-hydroxymethylcytosine (5hmC) (9). 5hmC was found to be approximately 40% as abundant as 5mC in the DNA of Purkinje cells of the cerebellum, while the overall levels of 5hmC in the mammalian genome are ~10% of the 5mC levels (10). Some research indicated that 5hmC might play an important role in the occurrence and development of AD and HD (11, 12). Gontier and colleagues have found that TET2 and 5hmC levels are decreased in the elderly hippocampus (13). Our previous study demonstrated the upregulation of TET2 in the cellular and animal model of PD, and further results indicated that TET2 knockdown attenuates 1-methyl-4-phenyl-1,2,3,6-tetrahydropyridine (MPTP)-induced behavioral impairments in mice (14). Another recent study also confirmed that, patients with PD exhibit an epigenetic and transcriptional upregulation of TET2, while TET2 depletion in a neuronal cell model results in cytosine modification changes that are reciprocal to those observed in PD neurons (15). Therefore, the widespread epigenetic dysregulation of enhancers in the PD cellular and animal model may be partly mediated by an increased TET2 expression or its activity. Suppressing the protein level or enzyme activity of TET2 *in vivo* has a neuroprotective effect and may have a substantial therapeutic potential for PD.

Other PD associated genes also seem to be regulated by histones modifications. There are higher levels of histone acetylation in the midbrain of dopaminergic (DA) neurons from PD patients compared to the controls (16). Silence information regulator 1 (SIRT1) is a nicotinamide adenine dinucleotide (NAD)-dependent histone deacetylase. It has been shown to regulate a variety of physiological and pathological processes, including metabolism, cell proliferation, cell differentiation, inflammatory immune response, oxidative stress, cell apoptosis, and other processes (17). In the adult brain, SIRT1 can modulate synaptic plasticity and memory formation (18). Singh et al. found that the enzymatic activity of SIRT1 is disturbed in patients with PD, which may make these patients particularly susceptible to neurotoxin-induced neuronal damage (19). Based on the neuroprotective effects of SIRT1 on PD, many *in vitro* and *in vivo* experiments have demonstrated that some SIRT1 activators, notably resveratrol (RV), have potential neuroprotective effects against DA neuronal damage caused by various neurotoxins (20), but the specific molecular mechanism underlying its action requires further elucidation. Our team has also done some experiments to verify the protective effects of SIRT1 on PD, and discovered some neuroprotective pathways regulated by SIRT1 (21–24). Yet, whether TET2 is also regulated by SIRT1 in PD remains unclear.

A recent study of hematopoietic stem/progenitor cells (HSPC) in myelodysplastic (MDS) syndromes showed that the expression of SIRT1 and TET2 is down-regulated, and SIRT1 is fused to a lysine residue conserved in the TET2 catalytic domain, which enhances the methylation activity of TET2, inhibiting the abnormal proliferation of HSPC (25). Sun et al. recently reported that SIRT1 deacetylates TET2 in myelodysplastic syndrome stem and progenitor cells. Enhanced TET2 activity marked by 5hmC was observed in MDS CD34^+^ cells upon SRT1720 treatment (26). In this study, we investigated the role of these two epigenetic modifications in the pathogenesis of PD by exploring the relationship between SIRT1 and TET2.

## Materials and Methods

### Cell Culture and Transfection

SH-SY5Y neuroblastoma cells were cultured in Dulbecco's modified eagle's medium (DMEM, Gibco) containing 10% fetal calf serum (Gibco, Brazil) and grown in a CO_2_ incubator maintained at atmospheric oxygen levels and 5% CO_2_. Dissolved MPP^+^ (Sigma Aldrich) in phosphate buffered saline (PBS) to a storage concentration of 125 mM. Resveratrol (Sigma) was prepared in dissolved dimethyl sulfoxide (DMSO) at a stock of 25 mM. EX-527, SRT1720, CHX, MG-132, Calpeptin, Z-VAD, and Bobcat339 (Selleckchem) stocks were dissolved in DMSO at the concentrations of 10 mM,10 mM, 1 mg/ml, 10 mM, 10 mM, 50 mM, and 3 mM, respectively. They were both stored at −20°C. Resveratrol, EX-527, and SRT1720 were pretreated cells 12 h before specific administration.

TET2 knockdown by lentiviral delivery of a short hairpin RNA (shRNA) was achieved via lentiviruses expressing the green fluorescent protein (GFP) and shRNA-TET2. shRNA-TET2 containing human shTET2a and shTET2b. Sequences for human shTET2a and shTET2b were (5′-GGGTAAGCCAAGAAAGAAA-3′) and (5′-AAACAAAGAGCAAGAGATT-3′), respectively.

### CCK8 Cell Viability Assay

The CCK-8 assay kit (Cell Counting Kit-8; Biomake) was used to determine cell viability. SH-SY5Y cells were seeded into 96-well plates at a density of 5,000 cells/well with 100 μl of cell medium in each well. The cells were treated differently after they were firmly attached. After a certain processing time, 10 μl of CCK-8 working solution was added to each well to incubate for about 1 h at 37°C. The wells containing only the culture medium served as blanks. The absorbance value at 450 nm was measured using a microplate reader.

### Cell Cycle Assays

Cell cycle analysis was performed using a Cell Cycle and Apoptosis Analysis Kit (Beyotime). After treatment, the cells were washed with PBS and fixed in 70% pre-cooled ethanol at 4°C overnight. Thereafter, the cells were re-suspended in 500 μl of PBS containing 0.2 mg/mL RNase A, and then 50 μg/mL propidium iodide (PI) was added to stain the cells for 30 min in the dark at 37°C. The percentages of cells at different phases (G0/G1, S, and G2/M) of the cell cycle were counted using a flow cytometer.

### Western Blot Analysis

The samples were lysed using a cell lysis buffer along with protease inhibitors. Equal amount of the protein samples was first separated by SDS-PAGE and then transferred to polyvinylidene fluoride (PVDF) membranes. Membranes were blocked with 5% non-fat milk in tris-buffered saline Tween (TBST) for 1 h at room temperature and then incubated overnight at 4°C with specific primary antibodies: anti-rabbit TET2 (Abcam), anti-rabbit SIRT1, anti-rabbit p16INK4a, anti-mouse Tubulin, anti-mouse LaminB1, anti-mouse HA-tag (CST), anti-rabbit CDK4, anti-rabbit pRb, anti-rabbit Bax, anti-rabbit Bcl-2, and anti-mouse GAPDH (Proteintech, United States). Then, the PVDF membranes were washed with TBST for three times and 10 min each time. Membranes were incubated with the corresponding secondary antibody horseradish peroxidase (HRP) conjugated at 1:5,000 dilution in 5% non-fat milk/TBST for 1 h at room temperature. Then, the membranes were washed with TBST following the previous method. After washing three times, the bands were visualized by using the enhanced chemiluminescence (ECL) reagents and an x-ray machine. The densitometric values of the bands were measured using the Image J software.

### Quantitative Real-Time Reverse Transcription Polymerase Chain Reaction (qRT-PCR)

SH-SY5Y cells after specific treatment were washed with PBS. Total RNA in cells was extracted using the Trizol reagent (Invitrogen Life Technologies, Carlsbad, CA, United States) according to the manufacturer's instructions. All RNA samples were purified and reverse transcribed into complementary deoxyribonucleic acid (cDNA), and quantitative PCR analysis was performed as described (27). The primer sequences used were:

TET2-F: 5′-ATTCTCGATTGTCTTCTCTAGTGAG-3′;
TET2-R: 5′-CATGTTTGGACTTCTGTGCTC -3′;
18sRNA-F: 5′-CAGCCACCCGAGATTGAGCA -3′;
18sRNA-R: 5′-TAGTAGCGACGGGCGGTGTG -3′;

### Nuclear and Cytoplasmic Separation

The cells were planted in a 10 cm dish. Two dishes of cells were needed. When the cells are full, the culture medium was discarded, and 1 × PBS was added to wash twice. The nuclear and cytoplasmic separation kit (Beyotime) was used to carry out the nuclear and cytoplasmic separation experiment. Refer to the kit instructions for specific experimental methods. Afterwards, the nucleoprotein uses LaminB1 as the control, and the cytoplasmic protein uses Tubulin as the control.

### Protein Immunoprecipitation

Add 500 μl of special cell lysis buffer for protein immunoprecipitation (50 mM Tris-HCL, 400 mM NaCl, 0.8% Triton-100X, PH7.5) to 10 cm dish cells. Put the dish on ice for 30 min, then scrape them and centrifuge at 12,000 rpm for 10 min (4°C). Take the supernatant into a new 1.5 ml centrifuge tube. Incubate with protein antibody at 4°C for 6 h, then add protein A/G agarose beads, and incubate overnight at 4°C. After that, use 1 × TBS to wash the beads three times, add 1 × loading buffer, heat at 95°C for 5 min, centrifuge, and take the supernatant for western blot test.

### Cell Ubiquitination Test

The cells were planted in a 10 cm dish, and Lipofectamine® 3000 (Catalog L3000-015) was used to transfect the cells with HA-Ub plasmid, 48 h later, add MG-132 (10 μM) for 6 h, then discard medium, add 500 ml cell lysate for protein immunoprecipitation (50 mM Tris-HCL, 400 mM NaCl, 0.8% Triton-100X, PH7.5) on ice for 30 min, and centrifuge at 12,000 rpm for 10 min (4°C). Take the supernatant into a new 1.5 ml centrifuge tube and incubate with anti-rabbit TET2 (Abcam) at 4°C for 6 h, then add protein A/G agarose beads, and incubate overnight at 4°C. Wash the beads three times with 1 × TBS, add the loading buffer, heat at 95°C for 5 min, centrifuge, and take the supernatant for western blot detection.

### Cellular Immunofluorescence

Seed the cells in a six-well plate covered with glass slides to a density of 60–80%. After the cells adhere to the wall, discard the culture medium and wash them with 1 × PBS, and add 1 ml of frozen methanol to fix at room temperature for 5 min. Then, add 1 ml of 1 × PBS to wash for 5 min, repeat twice, then add 1% Triton X-100 1 ml to break the membrane at room temperature. Wash with 1 × PBS for 5 min. After washing twice, add 1 mL of 1% BSA at room temperature for 30 min. After the cells and the primary antibody anti-rabbit TET2 (Abcam, 1:100) or anti-rabbit 5hmc (CST, 1:100) were co-incubated for 30 min at 37°C, wash the cells with 1 × PBS for three times, the corresponding secondary antibody Alexa-Fluor 594-conjugated anti-rat secondary antibody (Molecular Probes, diluted 1:100 in PBS) was incubated for 30 min at 37°C in the dark, dripped “prolong diamond antifade mountant with DAPI” (Invitrogen) at last. Finally, use the Zeiss LSM 780 to take pictures, and use the ZEN 2010 software (Zeiss) to analyze the pictures.

### Statistical Analysis

Data were presented as mean ± SD and each experiment was performed at least three times including the biological and technical replicates. The differences were evaluated using *t*-tests or two-way ANOVA using the GraphPad Prism software version 5. *P* < 0.05 were considered statistically significant.

## Results

### Alleviation of MPP^+^-Induced Injury of SH-SY5Y Cells by Resveratrol

**Figure 1:**
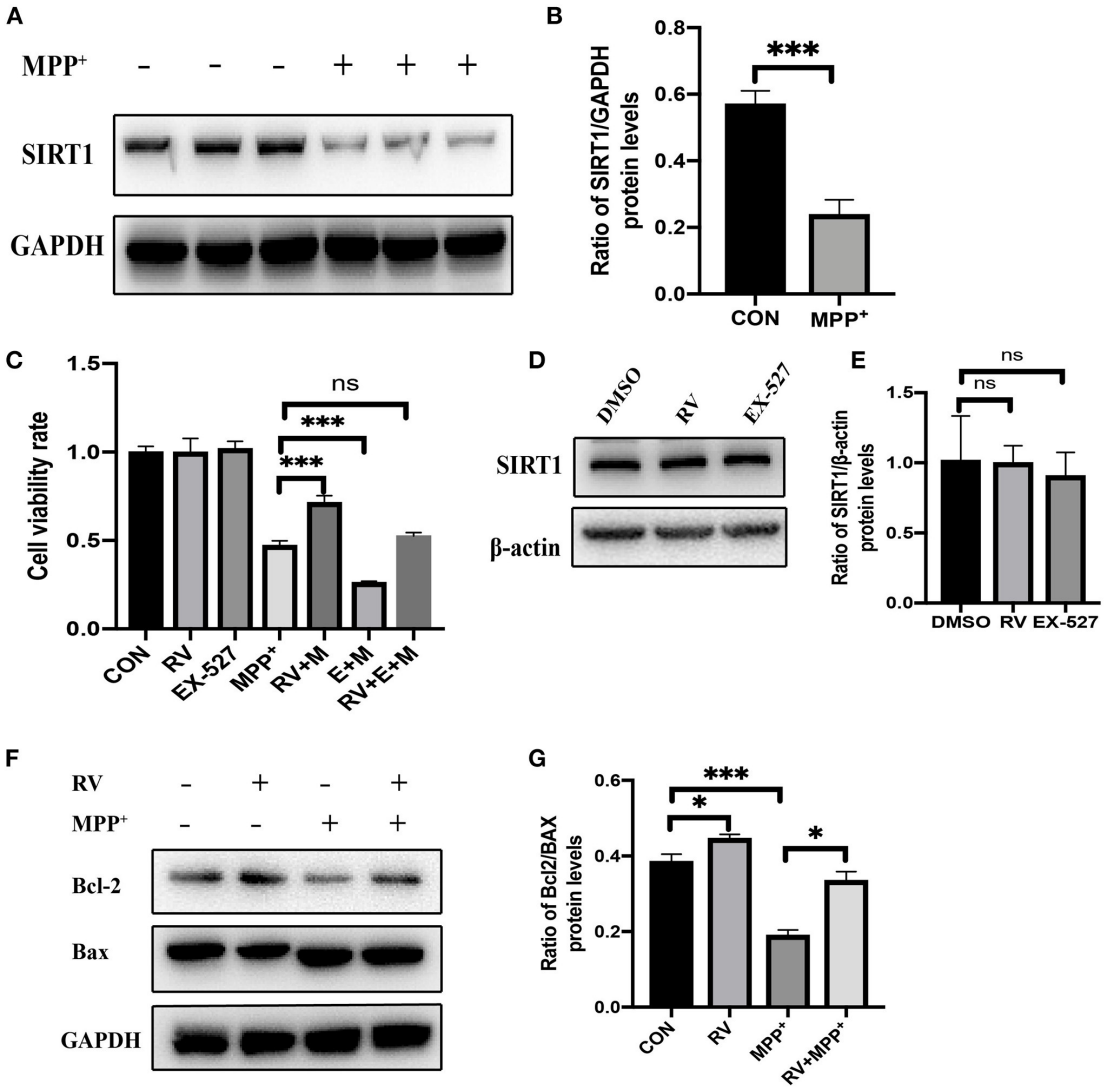
Alleviation of MPP^+^-induced injury of SH-SY5Y cells by resveratrol. **(A,B)** SH-SY5Y cells were treated with MPP^+^ (2.5 mM) for 24 h and the protein level of SIRT1 was detected via western blot. **(C)** SH-SY5Y cells were treated with RV (resveratrol 25 μM), EX-527(10 μM) 12 h before M (MPP^+^, 2.5 mM) treated and then co-treated with two or three of them for 24 h, then proliferation inhibition rates were examined by the CCK-8 method. **(D,E)** SH-SY5Y cells were treated with RV (resveratrol 25 μM), EX-527(10 μM) for 24 h and the protein level of SIRT1 was detected via western blot. **(F,G)** SH-SY5Y cells were treated with MPP^+^ (2.5 mM), RV (25 μM) separately, or were treated with RV 12 h before MPP^+^ and then co-treated with MPP^+^ for 24 h. Western blot and quantification of protein was performed to detect apoptotic proteins. Data are shown as the mean ± SD (*n* = 3); *P < 0.05, ***P < 0.001.

To determine whether SIRT1 was altered in the MPP^+^ treated cells, the protein levels of SIRT1 were detected by western blot following MPP^+^ treatment. SIRT1 was significantly decreased after MPP^+^ treatment at 24 h in our current study (Figures 1A,B). Next, we checked the effect of RV on cell viability and found that when cells were pre-treated with RV for 12 h, MPP^+^-induced cell death was dramatically attenuated (Figure 1C). SH-SY5Y cells were treated with RV or EX-527 for 24 h and the protein level of SIRT1 was detected via western blot, the results showed that RV and EX-527 did not affect the protein level of SIRT1 (Figures 1D,E). To determine whether SIRT1 is involved in the neuroprotective effect of RV, we used EX-527 (specific inhibitor of SIRT1) to treat SH-SY5Y cells. The results showed that EX-527 may aggravate the cell damage caused by MPP^+^. Meanwhile, when the cells were treated with both EX-527 and RV, EX-527 can abolish the rescue effect of RV from MPP^+^ toxicity (Figure 1C). The ratio of B-cell lymphoma 2 protein (Bcl-2)/Bcl-2-associated X protein (Bax) protein regulates the sensitivity of cells to apoptosis: the higher this ratio is, the less sensitive the cells are to apoptosis. Our results also showed that pre-treatment with RV reversed the neurotoxicity of MPP^+^ by promoting Bcl-2/Bax ratio, indicating that RV can reduce SH-SY5Y cells damage caused by neurotoxin through inhibiting apoptosis (Figures 1F,G). Taken together, our results suggested that RV decreased the cell death and apoptosis induced by MPP^+^, and this effect of RV was SIRT1-dependent.

### The Enzyme Activity of SIRT1 Is Negatively Correlated With the Protein Level of TET2

**Figure 2:**
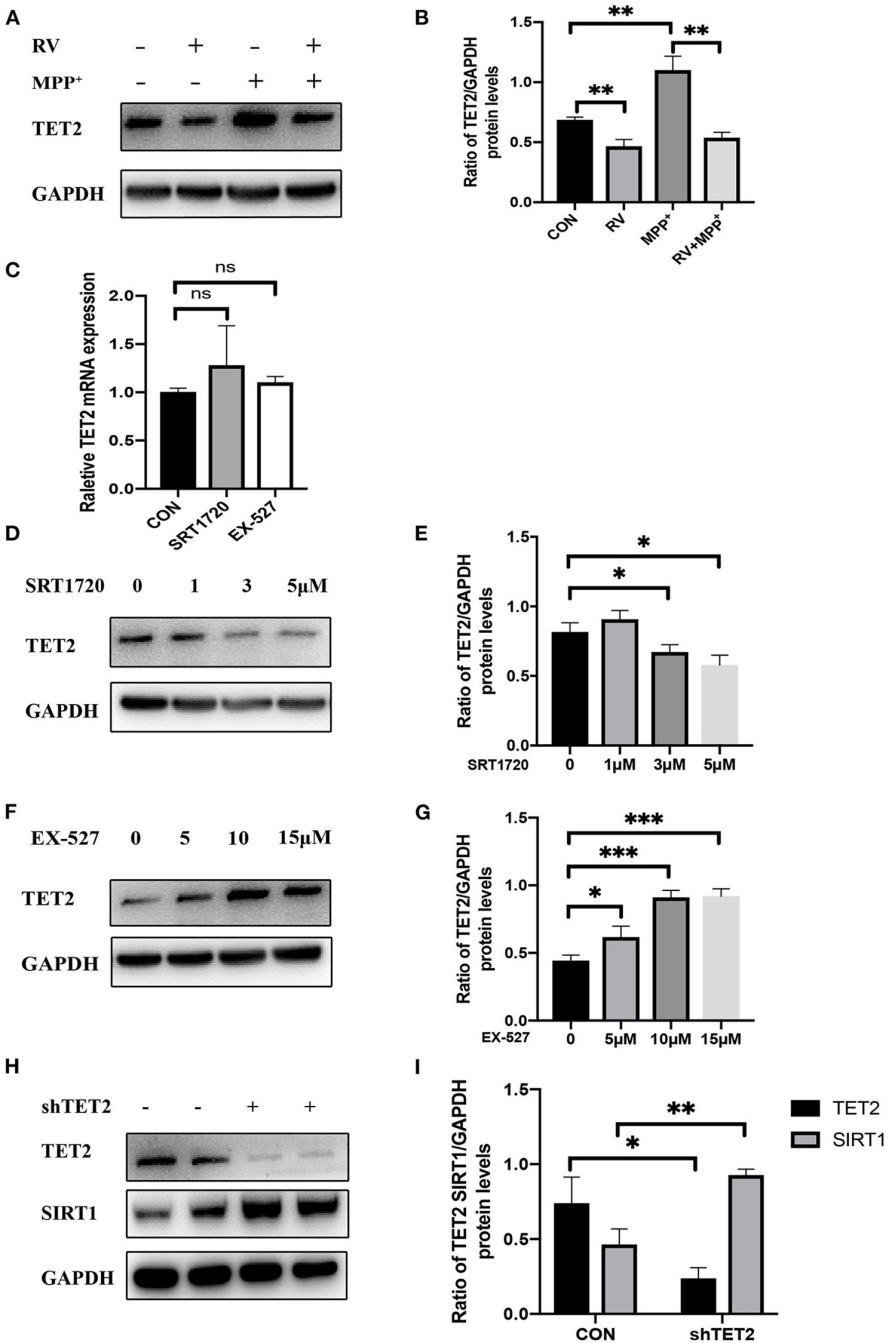
The enzyme activity of SIRT1 is negatively correlated with the protein level of TET2. **(A,B)** SH-SY5Y cells were treated with MPP^+^ (2.5 mM), RV (resveratrol 25 μM) separately or were treated with RV 12 h before MPP^+^ and then co-treated with MPP^+^ for 24 h. Western blot was used to detect the protein level of TET2. **(C)** qRT-PCR assay was used to test the mRNA level of TET2 in SH-SY5Y cells after SRT1720 (5 μM) or EX-527 (10 μM) were treated for 24 h. **(D–G)** SH-SY5Y cells were treated with a different dose of SRT1720 or EX-527 for 24 h to examine the protein level of TET2. **(H,I)** SH-SY5Y cells were transfected with shTET2 or shNC for 48 h. Western blot was used to detect the protein level of TET2 *P < 0.05, **P < 0.01, ***P < 0.001.

We divided SH-SY5Y cells into CON (control) group, RV treatment group, MPP^+^ treatment group, and RV and MPP^+^ co-treatment group. It showed that RV can inhibit the up-regulation of TET2 expression in DA neurons caused by MPP^+^ (Figures 2A,B). In order to more accurately study the regulatory relationship between SIRT1 and TET2, we treated SH-SY5Y cells with SIRT1 specific agonist SRT1720 and inhibitor EX-527, neither agonists nor inhibitors of SIRT1 affect the mRNA level of TET2 (Figure 2C). However, the protein expression of TET2 was significantly reduced after different concentrations of SRT1720 treatment (Figures 2D,E); while different concentrations of EX-527 induced the protein expression of TET2 significantly (Figures 2F,G). The above results indicate that the enzyme activity of SIRT1 is negatively correlated with the protein level of TET2. We are also curious about whether changes in the TET2 protein expression affect the SIRT1 protein level, so we detect the protein level of SIRT1 after transfecting the cells with shTET2 lentivirus. The results show that the down-regulation of TET2 will also lead to the up-regulation of SIRT1 (Figures 2H,I). Taking these results into consideration, we think that TET2 is significantly increased during the development of PD, and the activation of SIRT1 induced by RV can ameliorate the increase of TET2 in PD.

### Changes in Enzyme Activity of SIRT1 Affected the Functionality of TET2

**Figure 3:**
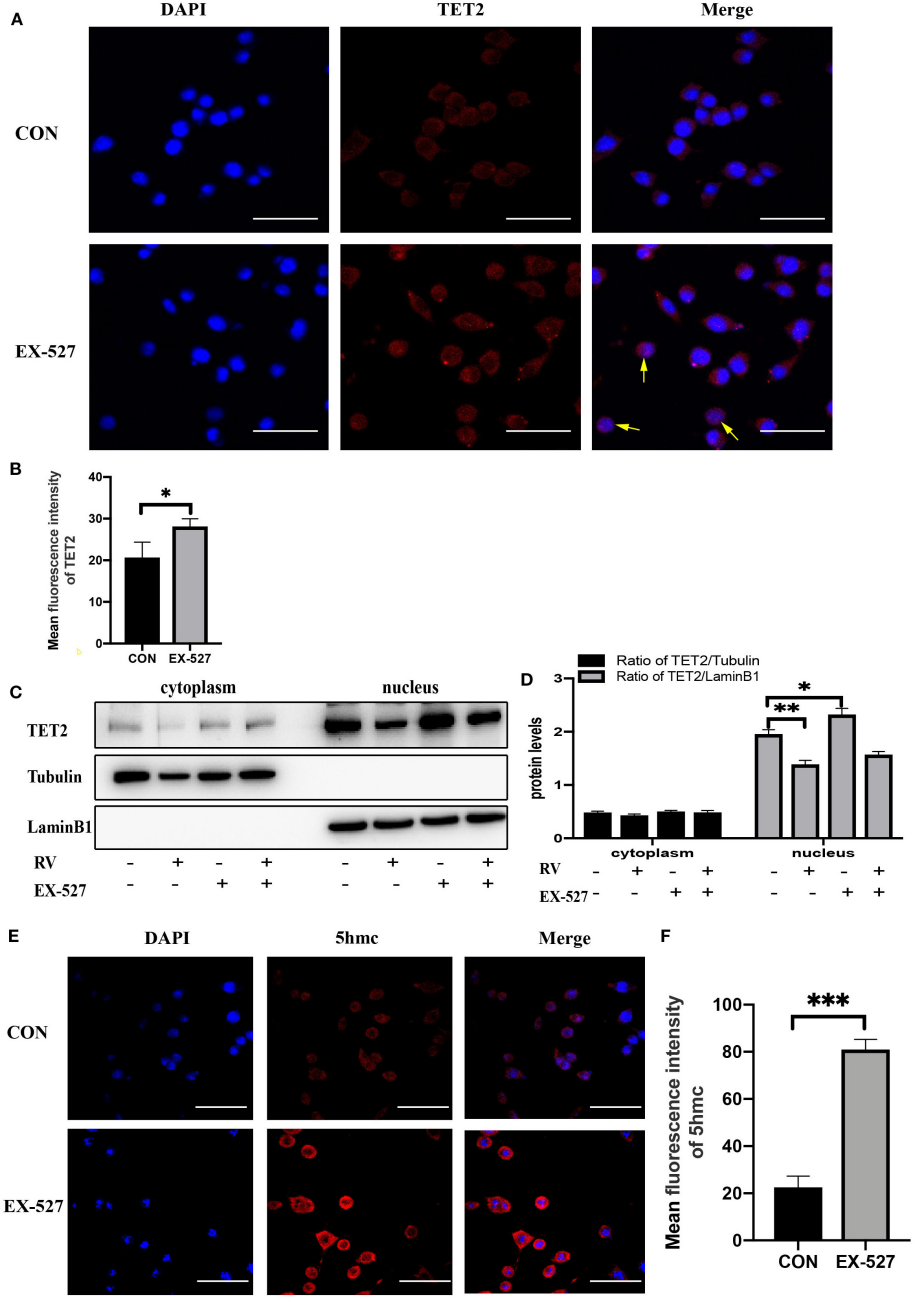
Changes in the enzyme activity of SIRT1 affected the functionality of TET2. **(A,B)** SH-SY5Y cells were treated with EX-527 (10 μM) for 24 h, and then immunofluorescence staining was performed to quantify the relative level of TET2, the yellow arrow refers to obvious nuclear transfer of TET2. Red: TET2; blue: DAPI counterstaining of DNA. The fluorescence intensity of the red fluorescent was measured using the Image J software. Shown are average values with SD of triplicated experiments. Scale bar = 100 μm *P < 0.05. **(C)** Nuclear and cytoplasmic protein level of TET2 after specific administration. **(D)** The quantification of the ratio of TET2/Tubulin or TET2/LaminB1. Data are shown as the mean ± SD (*n* = 3); *P < 0.05, **P < 0.01. **(E,F)** Immunofluorescence staining and quantification of 5hmc level after treatment with EX-527 (10 μM) for 24 h. Red: 5hmc; blue: DAPI. The fluorescence intensity of the red fluorescent was measured using the Image J software. Shown are average values with SD of triplicated experiments, Scale bar = 100 μm ***P < 0.001.

Since TET2 needs to interact with DNA binding partners to regulate gene expression in the nucleus (28, 29), we test whether SIRT1 activity affects the entry of TET2 into the nucleus and the level of 5hmc in SH-SY5Y cells. As shown in Figures 3A,B, immunofluorescence staining of TET2 (red) was conducted. Merged images showed that TET2 was both distributed in the cytoplasm and nucleus. After SIRT1 inhibition by EX-527, the protein level of TET2 increased and mainly accumulated in the nucleus. To further verify this phenomenon, we carried out nuclear and cytoplasmic separation of SH-SY5Y cells. Similarly, RV significantly decreased the level of TET2, whereas the inhibition of SIRT1 by EX-527 reversed the effect of RV on TET2 abundance. In addition, the above changes of TET2 were more significant in the nucleus than in the cytoplasm (Figures 3C,D). Meanwhile, we tested the level of 5hmc (red) in SH-SY5Y cells to prove the changes of enzyme activity of TET2. We found that the level of 5hmc was dramatically increased after EX-527 treatment (Figures 3E,F), illustrating the functional change of TET2.

### Deacetylation of TET2 Induced by SIRT1 Promotes TET2 Degradation via the Ubiquitin-Proteasome Pathway

**Figure 4:**
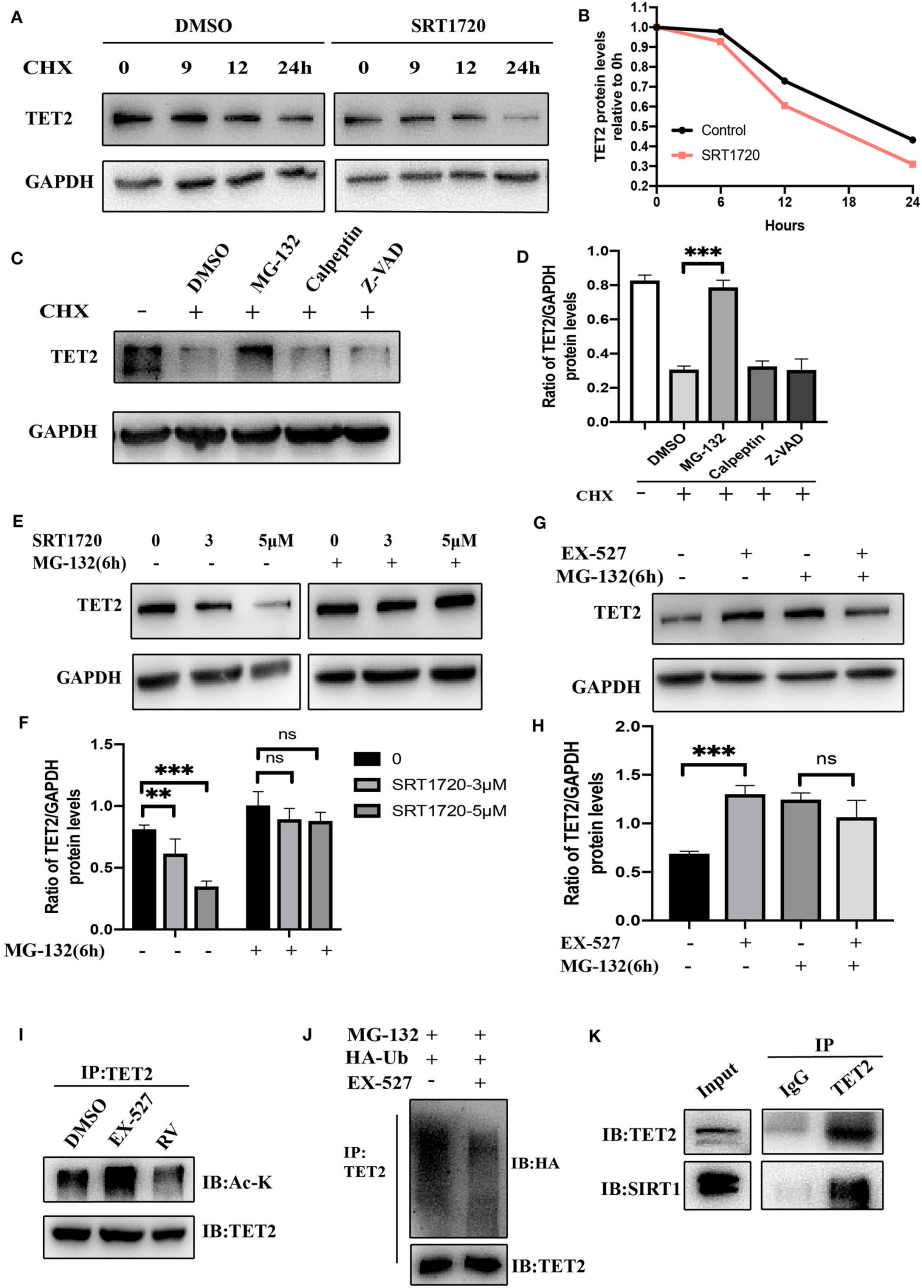
Deacetylation of TET2 induced by SIRT1 promotes TET2 degradation via the ubiquitin–proteasome pathway. TET2 deacetylation induced by SIRT1 promotes its degradation via the ubiquitin–proteasome pathway. **(A)** SH-SY5Y cells were pretreated with SRT1720 (5 μM) or DMSO for 12 h and then subjected to CHX (cycloheximide, 100 μg/ml) treatment for the indicated time. The protein level of TET2 was detected via western blot. **(B)** The trend of TET2 protein level was quantified by the Image J. **(C,D)** SH-SY5Y cells were treated with CHX (100 μg/ml) and various proteolysis inhibitors: MG-132 (10 μM), Calpeptin (10 μM), Z-VAD (50 μM) for 24 h. Western blot analysis and quantification of TET2 protein abundance was evaluated. **(E–H)** SH-SY5Y cells were treated with SRT1720 at various concentrations, or EX-527 at 10 μM for 24 h. MG-132 (10 μM) was added to medium 6 h before cells were collected. Western blot analysis and quantification of TET2 protein abundance was evaluated. **(I)** SH-SY5Y cells were treated with 10 μM of EX-527 or 25 μM of RV (resveratrol) for 24 h. CO-IP was used to detect endogenous acetylated TET2. **(J)** SH-SY5Y cells were transfected with HA-Ub for 24 h and then treated with DMSO or EX-527 for 24 h, MG-132 10 μM was added to the medium 6 h before cells were collected. Polyubiquitylation levels of TET2 proteins were detected by Western blot at last. **(K)** CO-IP was used to examine the interaction between TET2 and SIRT1. IP, immunoprecipitation; IB, immunoblotting. Data are shown as the mean ± SD (*n* = 3); **P < 0.01, ***P < 0.001.

From the above experimental results, we know that the enzyme activity of SIRT1 will not affect the transcription of TET2 but affect its protein level, so we further explore whether SIRT1 will affect the degradation of TET2. Inhibition of protein synthesis with cycloheximide (CHX) demonstrated that TET2 is a rather stable protein in SH-SY5Y cells, and there was still a small amount of protein 24 h after inhibiting its synthesis (Figure 4A). After SRT1720 treatment, TET2 was basically completely degraded at 24 h in SH-SY5Y cells (Figures 4A,B), suggesting that an increased SIRT1 enzyme activity destabilizes the TET2 protein. It has been previously reported that the stability of TET proteins may be regulated by degradation pathways mediated by caspase and calpains (30). Recent findings indicate that TET2 protein may be primarily regulated through the ubiquitin-proteasome pathway in A2780, HCT116 cells (31). In our study, we found that in SH-SY5Y cells, TET2 is stabilized by the proteasome inhibitor MG-132, but not by calpains or caspase inhibitors (Figures 4C,D). In order to explore whether SIRT1 affects the protein level of TET2 through the ubiquitinated proteasome pathway, we treated cells with different concentrations of SRT1720 (Figures 4E,F) and 10 μM of EX-527 (Figures 4G,H), and added MG-132 6 h before harvesting the cells. We found that with the participation of MG-132, the effect of SIRT1 enzyme activity on the TET2 protein abundance basically disappeared. In addition, EX-527 treatment, while increasing TET2 acetylation (Figure 4I), significantly reduces TET2 ubiquitination (Figure 4J). Finally, the TET2 protein that enters the ubiquitinated proteasome pathway for degradation is reduced, and the total amount of TET protein increases after EX-527 treated. Consistent with this result, the protein interaction between endogenous SIRT1 and TET2 was readily detected in SH-SY5Y cells (Figure 4K).

### SIRT1 Influenced Neuron Growth and Cell Cycle via TET2

**Figure 5:**
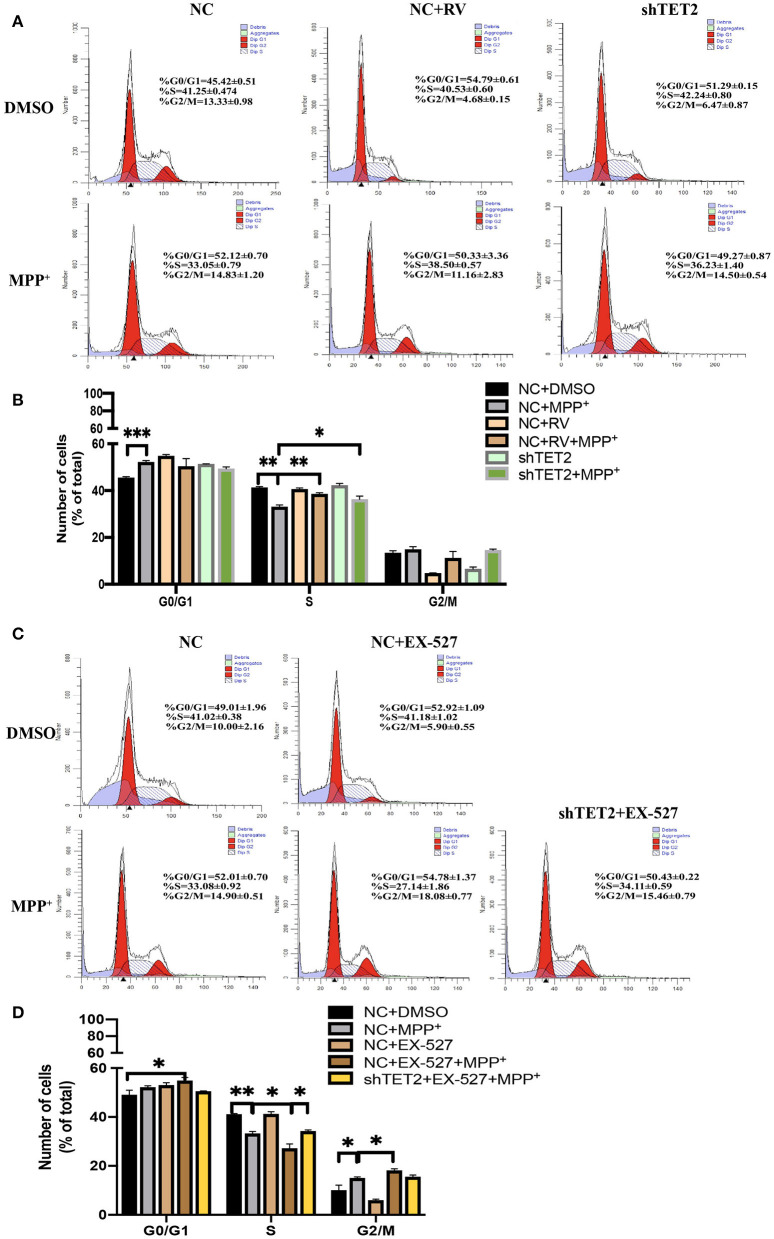
SIRT1 influenced neuron growth and cell cycle via TET2. **(A,B)** SH-SY5Y cells were treated with 25 μM of RV (resveratrol) for 24 h or transfected with shTET2 for 48 h. shNC and DMSO was used as control. Cell cycle analysis by PI/RNAase was conducted. **(C,D)** SH-SY5Y cells were treated with 10 μM EX-527 for 24 h accompanied with MPP^+^ (2.5 mM) or not. DMSO was used as control. Cell cycle analysis by PI/RNase was conducted. The cells have been transfected with shTET2 or shNC for 24 h before different drug treatment. Data are shown as the mean ± SD (*n* = 3); *P < 0.05, **P < 0.01.

Previous data from our laboratory showed that while rotenone blocks cell proliferation by arresting cells at the G0/G1 phase, SIRT1 promotes cell cycle progression to the S phase, thus contributing to cell proliferation (21). Moreover, TET2 also influenced the cell cycle (14). In the present study, incubation with MPP^+^ abrogated the S cell cycle progression in SH-SY5Y cells, with a significant decrease of cells in the S phase and a subsequent increase in cells in the G0/G1 and G2/M phases. Furthermore, this effect of MPP^+^ was noticeably weaker after RV treatment and TET2 knockdown (Figures 5A,B). When the SIRT1 enzyme activity was inhibited by EX-527 while MPP^+^ treated, the proportion of cells in the S phase is less than the cells treated with MPP^+^ alone. In addition, a subsequent increase in cells in the G0/G1 and G2/M phases were more significant. These results indicate that EX-527 exacerbates the cell cycle arrest caused by MPP^+^. Next, we inhibited the activity of SIRT1 enzyme in the cell while reducing TET2, and the aggravation of cell cycle arrest caused by EX-527 was reduced (Figures 5C,D). Therefore, we believe that SIRT1 is likely to reduce cell cycle arrest caused by neurotoxins at least in part by regulating TET2.

### The CDKN2A/p16INK4a-CDK4-pRB Signaling Pathway Is Involved in the Effect of TET2 on the Cell Cycle in PD Cellular Model

**Figure 6:**
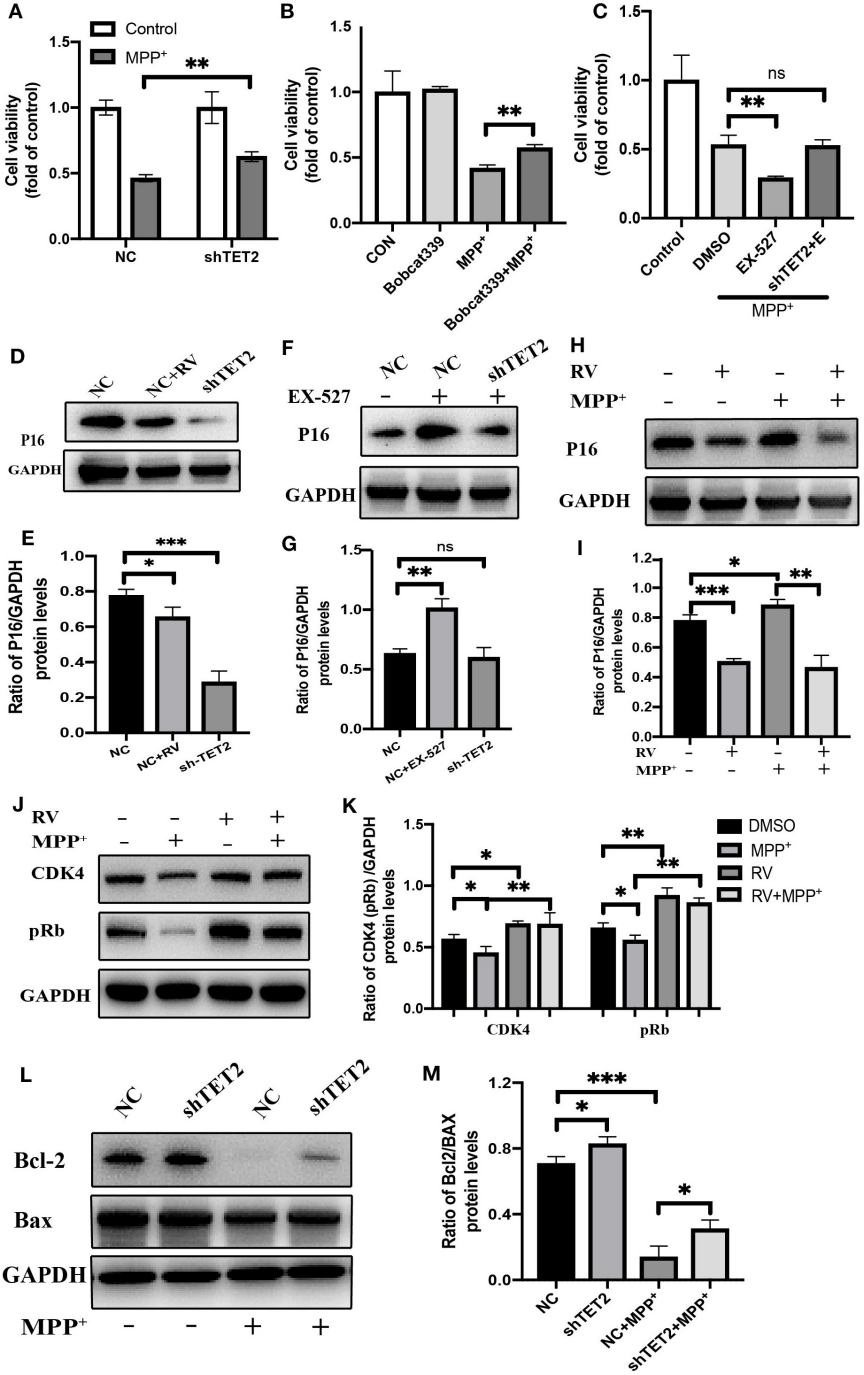
The CDKN2A/p16INK4a-CDK4-pRB signaling pathway is involved in the effect of TET2 on the cell cycle in PD cellular model. **(A,C)** The cells have been transfected with shRNA or NC for 24 h before different drug treatment. Proliferation inhibition rates was examined by the CCK-8 method. **(B)** Effects of TET2 inhibitor Bobcat339 on proliferation was detected by a CCK-8 assay. Bobcat339 (3 μM) was preincubated with cells for 12 h before MPP^+^ (2.5 mM) treatment. **(D,E)** SH-SY5Y cells were transfected with shTET2 or shNC for 24 h and then treated with RV (resveratrol 25 μM) **(D,E)**, or EX-527**(F,G)** for 24 h then western blot was used to detect the protein level of p16 (p16INK4a). SH-SY5Y cells were treated with MPP^+^ (2.5 mM), RV (25 μM) separately for 24 h or were treated with RV for 12 h before MPP^+^ and then co-treated with MPP^+^ for 24 h. Western blot and quantification of protein was performed to detect p16 **(H,I)** CDK4, and pRb **(J,K)**. **(L,M)** The SH-SY5Y cells have been transfected with shRNA or shNC for 24 h before MPP^+^ (2.5 mM) treatment. Western blot and quantification of protein was performed to detect apoptotic proteins. Data are shown as the mean ± SD (*n* = 3); *P < 0.05, **P < 0.01, ***P < 0.001.

Through the above experimental results, we see that SIRT1 can regulate TET2 to influence the cell cycle. Next, we use the CCK8 experiment to observe whether the SIRT1-TET2 pathway will ultimately affect the cell death induced by MPP^+^. In Figure 6A, knockdown of TET2 can weaken the damage of MPP^+^ to cells and increase cell viability. In addition to down-regulating the TET2 protein expression, we also used the TET2 enzyme activity inhibitors Bobcat339 to treat SH-SY5Y cells, and we observed that the decrease in the TET2 enzyme activity can also protect cells from neurotoxin damage (Figure 6B). When SIRT1 enzyme activity is inhibited, SH-SY5Y cells are more sensitive to the toxicity of MPP^+^. However, after knocking down TET2, the phenomenon of increased susceptibility of cells to MPP^+^ caused by EX-527 basically disappeared (Figure 6C).

Our group found that after MPP^+^ treatment, the hydroxymethylation peak of cyclin-dependent kinase inhibitor 2A (CDKN2A) gene promoter region increased by five times compared with the control group. Further research indicated that CDKN2A expression products p14ARF and p16INK4a were also significantly up-regulated, this upregulation was reversed by the knockdown of TET2 (14). CDKN2A is considered to be an important tumor suppressor gene, and it is frequently mutated or deleted in many tumors. It encodes two proteins: p16INK4a and p14ARF. Among them, the specific binding of the p16INK4a protein to cell cycle-dependent kinase 4 (CDK4) or CDK6 induces an allosteric conformational change in these proteins and inhibits the formation of the complex between CDK4 or 6 and cyclin D (32). The lack of this complex formation maintains the retinoblastoma protein (Rb) in its hypo-phosphorylated and growth-suppressive states. This leads to the induction of the G1 phase cell cycle arrest (33).

Western blot results showed that p16INK4a was decreased after RV treated, consistent with shTET2 cells (Figures 6D,E). In contrast, the EX-527 treatment leads to an increase in the protein level of p16INK4a, and this increasing trend weakens with the decrease in TET2 expression (Figures 6F,G). After MPP^+^ treatment, p16INK4a was significantly up-regulated, and the expression of its downstream genes CDK4 and pRb were significantly down-regulated. The protein expression of p16INK4a, CDK4, and pRb in SH-SY5Y cells pretreated with RV was opposite to the above-mentioned trend (Figures 6H–K). As shown in Figures 6L,M, TET2 negatively regulates the expression of Bcl-2, and knocking down TET2 can partially restore the decrease in Bcl-2 caused by MPP^+^ treatment. Taking the above results into consideration, we hypothesized that the activation of SIRT1 by RV can reduce the cell cycle arrest of SH-SY5Y cells caused by neurotoxins through down-regulating the TET2-CDKN2A/p16INK4a-CDK4-pRB signaling pathway. On the other hand, SIRT1 may directly regulate cell apoptosis through the effect of TET2 on the expression of Bcl-2 protein.

## Discussion

More than 60 years have passed since George Cotzias introduced high-dose levodopa therapy in 1957 (34), we are still living in the dopamine era when it comes to the treatment of PD. At present, the pathogenesis of PD has not yet been fully elucidated, lacking of neuroprotection or disease-modifying therapy. Medicines, deep brain stimulation, and rehabilitation training can only achieve partial relief, which cannot terminate or reverse the progression of PD. Therefore, it is urgent to explore new strategies for the treatment of PD. The pathogenesis of PD includes large-scale histone and DNA modifications, methylation and acetylation are both very important modification methods but the relationship between these two epigenetic changes is unclear.

Embryonic stem cells (ESCs) derived from SIRT6 knockout mice are skewed toward neuroectoderm development. This phenotype is associated with the depression of Oct4, Sox2, and Nanog, which in turn causes an upregulation of TET1/2 enzymes and elevated production of 5hmC (35). In human ovarian cancer cells and colon cancer cells, TET2 can bind to p300 (a histone acyltransferase) and be acetylated by p300, therefore, the hydroxymethylase activity of TET2 is enhanced. HDAC1/2 can deacetylate some lysine residues of TET2 and weaken its hydroxymethylase activity. HDACs inhibitors will delay the half-life of TET2 degradation by the proteasome, thereby increasing its protein level (31). In this study, we found for the first time that there is a negative correlation between the SIRT1 enzyme activity and TET2 protein level in the *in vitro* model of PD. The main mechanism may be that the increase of SIRT1 enzyme activity reduces the acetylation level of TET2, and then promotes TET2 degradation through the ubiquitin-proteasome pathway, resulting in a significant reduction in the TET2 protein level. SIRT1 also affects the content of TET2 in the nucleus and 5hmc in SH-SY5Y cells.

Neurons are post-mitotic cells which are defined as mature cells that have ceased replication and have permanently left the cell-cycle. However, cell cycle re-entry can be seen in post-mitotic cells under certain conditions (36), for instance, in neurons following insults to the CNS, such as stroke (37), mild cognitive impairment and early AD pathology (38), Huntington's diseases, as well as other neurodegenerative diseases (39, 40). Yet the same activation can also be seen in healthy controls and pathology-free neurons, though it was at lower levels (41). Restrained and limited cell cycle re-entry appears to be of use in aiding DNA repair in neurons. Hyperploid neurons also appear to be functionally active and maintain distal axonal connections (42), suggesting that there is likely an attractive advantage for maintaining hyperploid neurons and preventing apoptosis.

There are many aspects involved in the protective effect of SIRT1 on PD DA neurons, mainly including autophagy, mitochondrial function, inflammation, and apoptosis. Our previous findings provide evidence that RV represses p53 by activating SIRT1, especially through epigenetic modulation at the transcriptional level, thus inhibiting cell cycle arrest and apoptosis. In the work published by our team a few months ago, we explored a potential role for TET2 in the pathogenesis of PD for the first time and found that the downregulation of the TET2 significantly attenuated cell cycle arrest and apoptosis of SH-SY5Y cells via weakening the activity of CDKN2A in MPP^+^-induced SH-SY5Y cells. Consistent with these results, in the present study, when the SH-SY5Y cells were incubated with MPP^+^, the S phase of cell cycle was abrogated and cells in the G0/G1 and G2/M phases were increased. Furthermore, this effect of MPP^+^ was noticeably weaker after RV treatment and TET2 knockdown. Reducing the expression of TET2 can basically relieve the aggravation of cell cycle arrest caused by EX-527. Therefore, we believe that TET2 is also involved in the regulation of the cell cycle by SIRT1 in DA neurons. Besides, we observed the inhibitory effect of SIRT1 on apoptosis through the detection of Bcl-2 and Bax, and also found that RV treatment and TET2 knockdown can increase Bcl-2 protein level. Experimental results from Obexer et al. indicated that Bcl-2 protein and mRNA were markedly reduced after 48 h of p16INK4a expression in leukemia cells, suggesting that p16INK4a reconstitution at least in part exerts its effects via changes in the mRNA expression of Bcl-2 (43). Taken together, we think that SIRT1 may affect Bcl-2 through the regulation of the TET2-CDKN2A/p16INK4a pathway. In addition to regulating the cell cycle progression, which suppresses growth and induces cell cycle arrest and apoptosis of DA neurons in PD, TET2 inactivation in mice fully prevents nigral dopaminergic neuronal loss induced by previous inflammation. TET2 loss also attenuates transcriptional immune responses to an inflammatory trigger (15).

It is well-known that small molecules like resveratrol and synthetic SIRT1 activators were shown to activate the enzyme directly via an allosteric site adjacent to the catalytic domain (44). Although we discuss the neuroprotection effects of resveratrol, it may also hit non-SIRT1 neuronal targets to affect biological outcomes *in vivo*. Therefore, in order to more specifically verify the role of SIRT1, we used SRT1720 and EX-527 as well. Moreover, we found that the down-regulation of TET2 will also lead to the up-regulation of SIRT1 (Figure 2H). This phenomenon may be caused by a negative feedback regulation between TET2 and SIRT1, and TET2 may also affect the expression of SIRT1 through some signaling pathways, but more research is required to answer this question. Therefore, we should conduct research in the PD *in vivo* model to better understand the role of SIRT1 and TET2 in the pathogenesis of PD and the relationship between these two genes.

**Figure 7:**
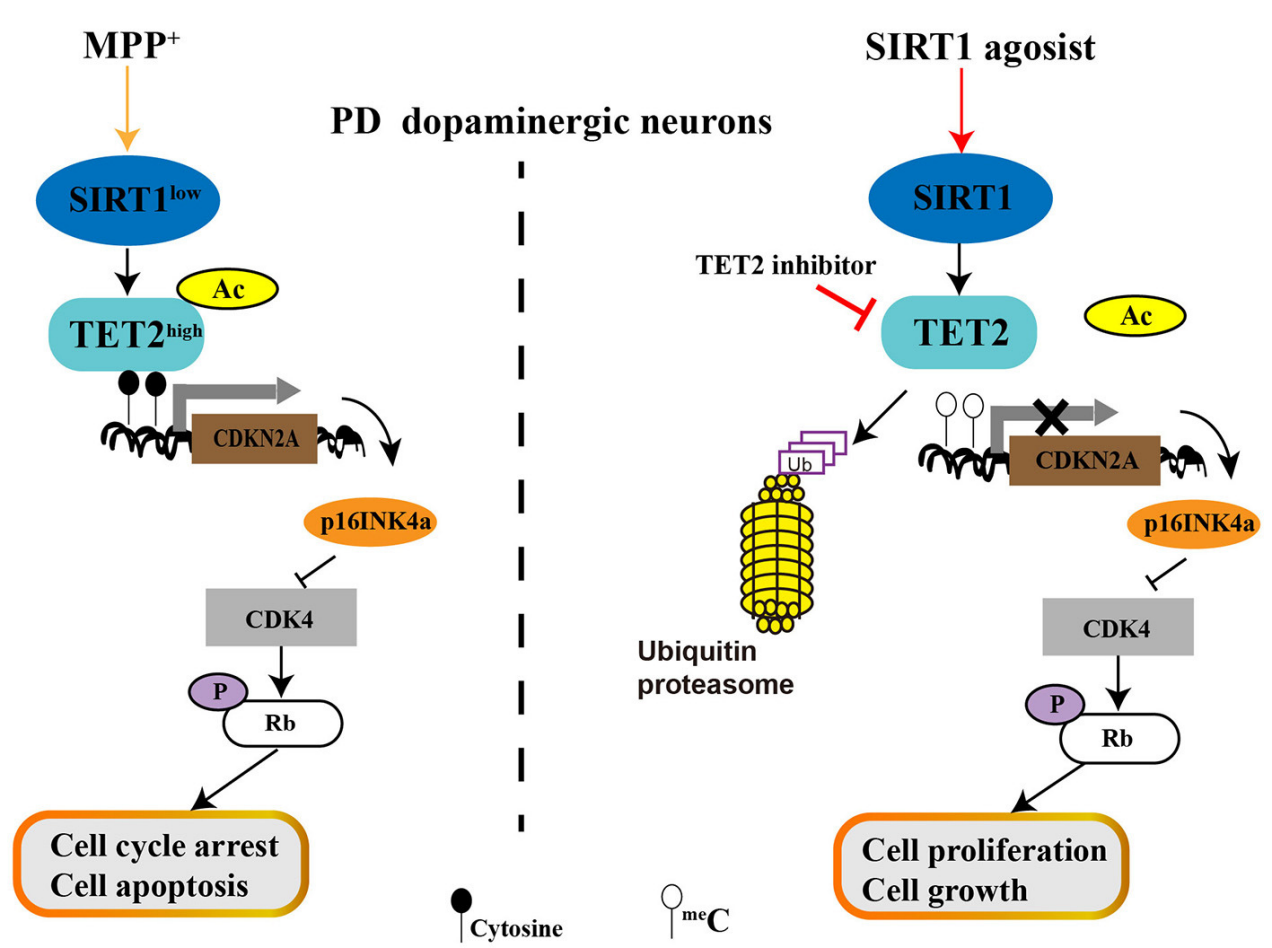
The role of SIRT1-TET2-CDKN2A**/**p16INK4a pathway in dopaminergic neurons. MPP^+^ treatment decreased SIRT1 expression and then promotes TET2 hyperacetylation, resulting in the increased stability of TET2 protein. Excess TET2 protein induce DNA demethylation and TET2 targeted gene expression, including CDKN2A. The elevated protein level of p16INK4a (One of the two proteins CDKN2A encodes) inhibit CDK4 and then reduce the pRb protein level, which allows cell cycle arrest and induces cell apoptosis of PD SH-SY5Y cells. Conversely, activation of endogenous SIRT1 by resveratrol (RV) leads to CDKN2A DNA hypermethylation due to the decreased TET2 protein levels, which relieves the inhibitory effect on CDK4 and upregulation of pRb, allowing cell proliferation and growth. Similar effects are observed for inhibition of endogenous TET2 enzyme activity with TET2 inhibitor.

Our finding showed that MPP^+^ treatment decreases SIRT1 expression and then promotes TET2 hyperacetylation, resulting in the increased stability of the TET2 protein. Excess TET2 protein induce DNA demethylation and TET2 targeted gene expression, include CDKN2A. The elevated protein level of p16INK4a (One of the two proteins CDKN2A encodes) inhibit CDK4 and then reduce the pRb protein level, which allows cell cycle arrest and induces cell apoptosis of SH-SY5Y cells. Conversely, activation of endogenous SIRT1 by RV leads to CDKN2A DNA hypermethylation due to the decreased TET2 protein levels, which relieves the inhibitory effect on CDK4 and upregulation of pRb, allowing cell proliferation and growth. Similar effects are observed for the inhibition of endogenous TET2 enzyme activity with TET2 inhibitor (Figure 7). With the clinical and therapeutic significance, regulating the SIRT1 expression or/and SIRT1 enzyme activity is a potential therapeutic strategy for PD, and the present study provides new possible mechanisms underlying its function. Our results further indicate that SIRT1 can affect cell cycle and apoptosis by regulating TET2 in PD progression, and targeted downregulation or inhibition of TET2 enzyme activity could be developed as a therapeutic intervention for PD, which warrants further investigation.

## Conclusions

In our study, we demonstrate that RV could attenuate MPP^+^ induced cell cycle arrest and cell apoptosis. The underlying mechanism may include the activation of the SIRT1-TET2-CDKN2A pathway. Activated endogenous SIRT1 by RV can downregulate TET2 acetylation, promoting TET2 degradation through the ubiquitinated proteasome pathway. Decreased TET2 protein level leads to CDKN2A DNA hypermethylation, the inhibitory effect of p16INK4a on CDK4 is relieved and pRb increases, allowing cell proliferation and growth. Inhibition of endogenous TET2 enzyme activity with TET2 inhibitor also has above effects. Therefore, the regulation of SIRT1-TET2-CDKN2A signaling pathway can be applied to the treatment and intervention of PD.

## Data Availability Statement

The original contributions presented in the study are included in the article/Supplementary Material, further inquiries can be directed to the corresponding authors.

## Author Contributions

Y-CW and HY provided fund support, revised the manuscript, and designed project ideas. TL revised the manuscript. XL conducted the experiments and wrote the manuscript. YF, T-TW, and S-JP provided experimental materials and assist XL in problem-solving.

## Conflict of Interest

The authors declare that the research was conducted in the absence of any commercial or financial relationships that could be construed as a potential conflict of interest.

## Abbreviations

PD: Parkinson's disease
AD: Alzheimer's disease
TET2: Ten-eleven translocation 2
SIRT1: Silence information regulator 1
RV: Resveratrol
5mC: 5-methylcytosine
5hmC: 5-hydroxymethylcytosine
Bax: Bcl-2-associated X protein
Bcl-2: B-cell lymphoma 2
MPP^+^: 1-methyl-4-phenyl-pyridinium ion
MPTP: 1-methyl-4-phenyl- 1,2,3,6-tetrahydropyridine
CDKN2A: cyclin dependent kinase inhibitor 2A
CDK4: cell cycle-dependent kinase 4
DA: dopaminergic
Rb: retinoblastoma protein.
